# Solvent-Free Enzymatic Synthesis of Dietary Triacylglycerols from Cottonseed Oil in a Fluidized Bed Reactor

**DOI:** 10.3390/molecules28145384

**Published:** 2023-07-13

**Authors:** Daniela Remonatto, Núbia Santaella, Lindomar Alberto Lerin, Juliana Cristina Bassan, Marcel Otávio Cerri, Ariela Veloso de Paula

**Affiliations:** 1 Department of Bioprocess Engineering and Biotechnology, School of Pharmaceutical Sciences, São Paulo State University (UNESP), Araraquara 14800-903, SP, Brazil; d.remonatto@unesp.br (D.R.); nubiasantaella@gmail.com (N.S.); juliana.bassan@unesp.br (J.C.B.); marcel.cerri@unesp.br (M.O.C.); 2 Department of Chemistry, Pharmaceutical and Agricultural Sciences, University of Ferrara (UNIFE), Via Luigi Borsari, 46, 44121 Ferrara, Italy; 3 State Center for Technological Education Paula Souza, Faculty of Technology of Barretos (FATEC), Barretos 14780-060, SP, Brazil

**Keywords:** lipase, enzyme, acidolysis, structured lipids, triacylglycerols

## Abstract

The synthesis of structured lipids with nutraceutical applications, such as medium-long-medium (MLM) triacylglycerols, via modification of oils and fats represents a challenge for the food industry. This study aimed to synthesize MLM-type dietary triacylglycerols by enzymatic acidolysis of cottonseed oil and capric acid (C10) catalyzed by Lipozyme RM IM (lipase from *Rhizomucor miehei*) in a fluidized bed reactor (FBR). After chemical characterization of the feedstock and hydrodynamic characterization of the reactor, a 2^2^ central composite rotatable design was used to optimize capric acid incorporation. The independent variables were cycle number (20–70) and cottonseed oil/capric acid molar ratio (1:2–1:4). The temperature was set at 45 °C. The best conditions, namely a 1:4 oil/acid molar ratio and 80 cycles (17.34 h), provided a degree of incorporation of about 40 mol%, as shown by compositional analysis of the modified oil. Lipozyme RM IM showed good operational stability (*k*_d_ = 2.72 × 10^−4^ h^−1^, *t*_1/2_ = 2545.78 h), confirming the good reuse capacity of the enzyme in the acidolysis of cottonseed oil with capric acid. It is concluded that an FBR configuration is a promising alternative for the enzymatic synthesis of MLM triacylglycerols.

## 1. Introduction

Lipases (triacylglycerol hydrolases, EC 3.1.1.3) hold a prominent position in the food, pharmaceutical, and chemical industries because of their high selectivity, catalytic efficiency, and operational stability [1,2]. These enzymes typically catalyze the hydrolysis of triacylglycerols into fatty acids and glycerol. Under non-aqueous conditions, however, lipases are capable of catalyzing esterification, transesterification, aminolysis, interesterification, and acidolysis reactions on a wide variety of substrates [3,4,5,6,7]. Another interesting characteristic of lipases is their ability to act on the different ester bonds of triacylglycerol, which makes it possible to control the incorporation and distribution of fatty acids in the glycerol backbone, a fundamental mechanism for the production of structured lipids. Structural modification can enhance the nutritional, technological, and functional properties of lipids, producing molecules with reduced energy content, immune regulatory functions [8,9], or improved fluidity, elasticity, melting point, or high-temperature behavior [10].

Lipozyme RM IM is a *sn*-1,3 regioselective triacylglycerol lipase from *Rhizmucor miehei*, immobilized on a macroporous anion exchange resin, produced by Novozymes S.A. [11]. Lipases that are *sn*-1,3 regioselectives typically do not exchange acyl groups at the *sn*-2 position due to steric hindrance [12], thus allowing the control of fatty acids incorporation at the *sn*-1 and *sn*-3 specific positions of the glycerol chain in the triacylglycerol structure [2,13]. Lipozyme RM IM was chosen as a biocatalyst in this study due to its selectivity, efficiency in dietary triacylglycerol synthesis, high operational stability, and higher activity towards medium-chain and long-chain fatty acids [9,14,15,16,17,18].

**Figure 1:**
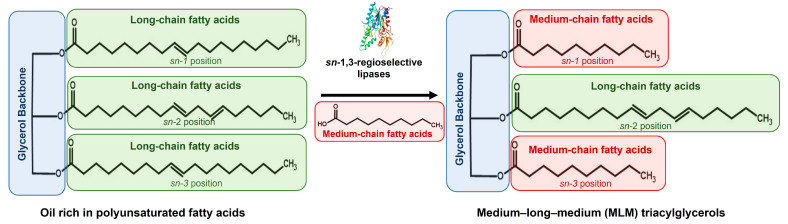
Synthesis of medium-long-medium (MLM) triacylglycerols by *sn*-1,3-regioselective lipases via acidolysis or interesterification.

Dietary triacylglycerols can be synthesized by *sn*-1,3-regioselective lipases via acidolysis or interesterification (Figure 1). These structured lipids have an energy content ranging from 5 to 7 kcal g^−1^, whereas conventional fats and oils provide 9 kcal g^−1^. Particularly noteworthy for their nutritional properties are triacylglycerol with a medium-long-medium (MLM) chain structure (i.e., triacylglycerols with medium-chain fatty acids at the *sn*-1 and -3 positions of glycerol and a long-chain fatty acid at the *sn*-2 position) (Figure 1). From a dietary point of view, the major advantage of MLM triacylglycerols is that they are rapidly metabolized as energy sources, bypassing storage in adipose tissues [9,10,19,20]. This mechanism aids in controlling lipid levels in the blood after a meal (postprandial triacylglycerols and plasma fatty acids), potentially contributing to body weight management [21].

New sources of triacylglycerols have been studied for technological applications, particularly those with high concentrations of long-chain polyunsaturated fatty acids, such as grape seed oil, which contains 58–78% linoleic acid [9,16]. Substrates with a high content of long-chain polyunsaturated fatty acids can be used for the synthesis of MLM triacylglycerols (Figure 1). Another rich source of polyunsaturated fatty acids is cottonseed oil. This plant oil is obtained by pressing cotton seeds, a byproduct of cotton (*Gossypium hirsutum*) generated by the textile industry [22]. Cottonseed oil contains about 50% polyunsaturated fatty acids, mainly linoleic acid (50.5–56.6%) [23]. Furthermore, cottonseed oil has great oxidative stability, attributed to its high antioxidant activity resulting from high concentrations of vitamin E [22,24,25].

In enzymatic synthesis, it is crucial to select adequate raw materials and reactor configurations [26]. Factors such as conversion efficiency, quality of final products, enzyme stability and reusability, and operating costs have a direct influence on the performance, productivity, and financial viability of the catalytic process [27]. Enzyme-improvement strategies include immobilization as, if well designed, immobilization can be a very powerful tool to improve enzyme stability and applicability [2,28]. Immobilization means combining the selectivity, stability, and kinetic properties of that enzyme with the mechanical properties of the carrier, maximizing the stability of both the physical and enzymatic properties of the biocatalyst [12,29]. In addition, the immobilization of the enzyme facilitates its recovery from the reaction medium, facilitating its reuse and, consequently, its application in various reactor configurations [30,31]. For this, the careful selection of the immobilization method and carrier matrix has fundamental importance [28].

The synthesis of MLM triacylglycerols can be performed both in batch, where the batch stirred tank reactor (BSTR) stands out as the primary method [9,21,32,33,34], and continuous mode, where the packed-bed reactor (PBR) stands out [16,35,36,37]. Another reactor configuration that can be employed for MLM-structured lipid synthesis is fluidized bed reactors (FBRs); however, this is less explored in the current literature. FBR systems are advantageous because they reduce changes in pressure at high feed flow rates, preventing the formation of concentration gradients [38]. Another interesting characteristic is that these reactors allow for the use of lower enzyme loads, as a free volume is required to maintain the enzyme and support suspension [39]. Due lower enzyme loading, the synthesis may require longer reaction times; however, sometimes it could be compensated by the fastest reaction caused by a less impeded mass transfer [10]. When it comes to viscous media, such as oils, fluidization minimizes clogging issues and reduces the pressure on the column matrix, which can be problematic in packed-bed reactors [40]. Compared to BSTRs, in FBRs the catalyst is subject to low mechanical stress, which makes this type of equipment an interesting application for enzyme catalysis reactions, as it improves the operational stability of these expensive biocatalysts [2,41]. Despite their operational advantages, FBR systems remain little explored. Thus, the application of fluidized bed reactors for the enzymatic synthesis of MLM triacylglycerols is a valuable contribution as it explores the use of these reactors as an alternative to the stirred-tank and fixed-bed reactors usually applied to lipid interesterification.

In light of the preceding information, this study aimed to synthesize MLM triacylglycerols by enzymatic acidolysis of cottonseed oil and capric acid (C10) using Lipozyme RM IM (lipase from *Rhizomucor miehei*) under solvent-free conditions in an FBR.

## 2. Results and Discussion

### 2.1. Characterization of Cottonseed Oil

The fatty acid profile of cottonseed oil is presented in Table 1. Fatty acid contents are similar to those reported in previous studies [42,43] and meet the requirements of the United Nations’ Food and Agriculture Organization [44]. Variations in the content of certain fatty acids may be due to factors such as seed variety and agroclimatic conditions [45]. Cottonseed oil was found to be composed of saturated and unsaturated fatty acids, with a predominance of unsaturated lipids (81.68%). The major component was the essential fatty linoleic acid (C18:2n6, 50.24%), followed by oleic acid (26.94%), palmitic acid (11.27%), and stearic acid (4.83%). The highly polyunsaturated fatty acids, linolenic and gamma-linolenic, were detected in relatively low amounts, 4.28 and 0.22%, respectively.

Linoleic (C18:2n6) and α-linolenic (C18:3n3) acids are essential fatty acids linked to several health benefits. These molecules serve as precursors to omega-3 and omega-6 polyunsaturated fatty acids, which are necessary for the adequate functioning of the immune system, development of the central nervous system, and maintenance of cardiovascular health [46,47,48]. Dietary intake of linoleic acid reduces carcinogenesis, atherosclerosis, and diabetes, in addition to contributing to bone formation and having anti-inflammatory properties [9,49,50]. Oils with a high content of oleic acid have greater oxidative and thermal stability, which improves shelf life [51]. Furthermore, oleic acid is an important precursor of oleoylethanolamide, a well-known satiety signaling molecule produced by the proximal intestine with a fundamental role in regulating energy metabolism and food intake [52]. Of note, long-chain (>12 carbon atoms), polyunsaturated, and essential fatty acids, such as those found in cottonseed oil, are key raw materials for the synthesis of MLM triacylglycerols [53,54].

Physicochemical analyses showed that the cottonseed oil used here had a peroxide value of 2.33 ± 0.431 mEq kg^−1^ and an acid value of 0.3 ± 0.01 mg KOH g^−1^. These results demonstrate that the oil meets the requirements for human consumption [44,55], which are as follows: the peroxide value should not exceed 10 mEq kg^−1^ and the acid values should not exceed 0.6 mg KOH g^−1^.

### 2.2. Density and Viscosity of Substrate Mixtures at Different Molar Ratios

Density and viscosity are important properties influencing the performance of reactor systems. These parameters should be measured prior to each reaction to ensure homogeneity among systems. The density of mixtures of cottonseed oil and capric acid at molar ratios of 1:2, 1:3, and 1:4 is presented in Table 2.

**Figure 2:**
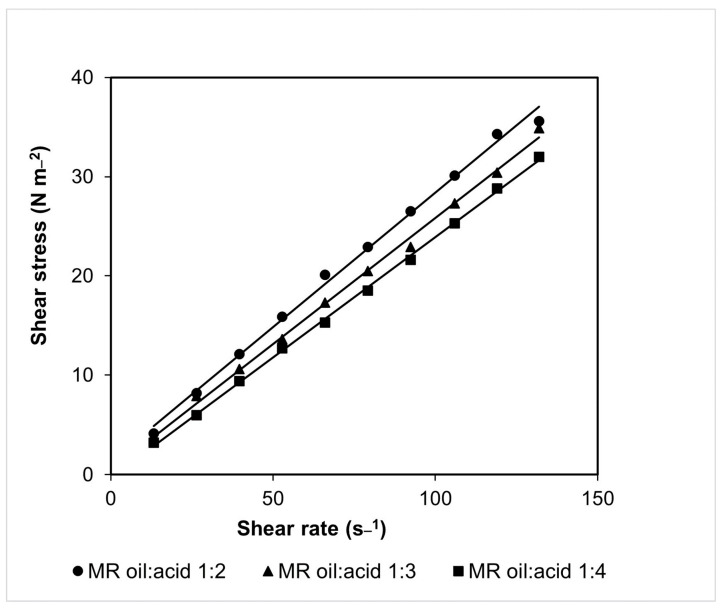
Shear stress as a function of shear rate for viscosity determination on media containing cottonseed oil and capric acid at molar ratios of 1:2, 1:3, and 1:4.

Density results did not differ (*p* > 0.05) between the three oil/acid molar ratios (1:2, 1:3, and 1:4). Thus, it was shown that the use of oil/acid molar ratios of 1:2 to 1:4 provided similar densities, not altering the reaction system. Figure 2 presents the curves of shear stress as a function of shear rate, which were used to determine viscosity according to oil/acid molar ratio.

Linearization of experimental data (Figure 2) resulted in Equations (1)–(3) and their correlation coefficients (R^2^) for each of the evaluated oil/acid molar ratios (1:2; 1:3; and 1:4, respectively).

y = 27.089x + 129.48 (R^2^ = 0.995) (1)

y = 25.387x + 45.394 (R^2^ = 0.9972) (2)

y = 24.221x − 32.195 (R^2^ = 0.999) (3)

All curves showed good linearity (R^2^ > 0.99), allowing estimation of viscosity using the angular coefficient. Viscosities of 27.089, 25.387, and 24.221 N s m^−2^ were obtained using cottonseed oil/capric acid molar ratios of 1:2, 1:3, and 1:4, respectively. The similarity of the viscosity values suggests that an increase in capric acid in the reaction medium would not affect the viscosity of the system, providing similar fluid flow rates in the column reactor. 

### 2.3. Hydrodynamic Characterization of the FBR System

#### 2.3.1. Minimum Fluidization Velocity

**Figure 3:**
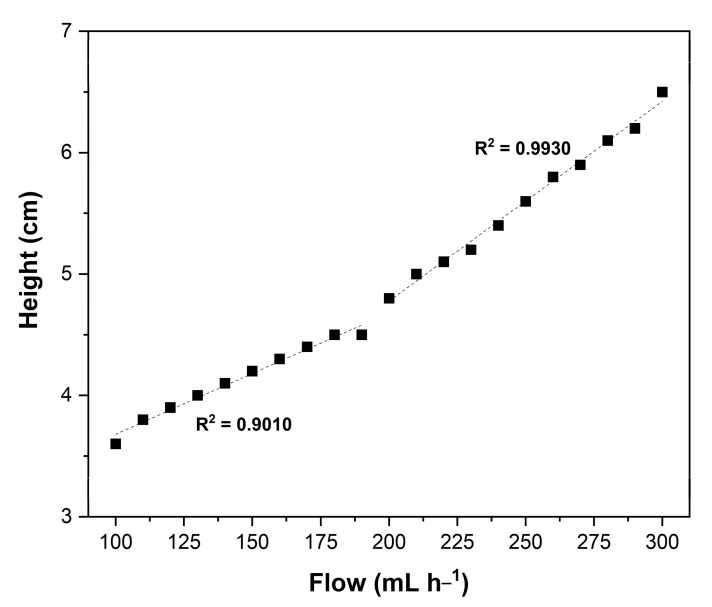
Feed flow rate of the reaction medium as a function of bed height.

The method described by Kunii and Levenspiel [56] was used for the determination of the minimum fluidization velocity. For this, a graph of bed height (cm) as a function of flow rate (mL h^−1^) was constructed (Figure 3). A change in the slope of the curve was observed, which can be represented by two distinct lines. According to Kunii and Levenspiel [43], the point at which the slope change occurs represents the minimum fluidization velocity. For the current experiment, the minimum fluidization velocity was found to be 200 mL h^−1^, confirming experimental observations. At this flow rate, complete fluidization of the biocatalytic bed, in addition to its expansion, was observed.

#### 2.3.2. Hydrodynamic Characterization of FBR

Hydrodynamic characterization of reactors is fundamental to assess the existence of preferential pathways. Experimentally, residence time distribution is determined by injecting a compound, called tracer, into the reactor at time zero and then measuring its concentration in the reactor effluent as a function of time. The tracer must be non-reactive, easily detectable, with physical properties similar to those of the reagent mixture, and completely soluble [57]. Here, we used Mix Verde, a fat-soluble dye, and constructed an analytical curve using the equation Abs = 0.9094C, with R^2^ = 0.9992, where C is the dye concentration (g L^−1^) and Abs is the absorbance (see Supplementary Material, Table S1 and Figure S1). 

Residence time distribution was determined after injection of the tracer into the base of the fluidized bed reactor, in the shortest time possible, using pulse injection [57]. The E(t) function was calculated (Equation (6)) using tracer concentration data. Using E(t), the mean residence time (Equation (7)) was found to be 12.80 min. Considering the volume of the support (2.04 mL), the useful volume of the reactor (57.65 mL), and the flow rate (300 mL h^−1^), the expected reaction time was 11.53 min (Equation (8)). 

In ideal reactors, with no preferential pathways, the mean residence time must coincide with the space time of closed systems [57]. The greater the number of preferential pathways, the greater the difference between mean residence time and space time (τ). In this study, the difference between residence time and space time was 10.0%, suggesting the presence of preferential pathways. Nevertheless, the difference between parameters was relatively low, considering that the fluidization behavior of particles of the immobilized derivative through the reaction medium did not significantly deviate from that of an ideal tubular flow. Therefore, particle velocity profiles were evenly distributed in the column reactor. 

### 2.4. Acidolysis of Cottonseed Oil in FBR

#### 2.4.1. Preliminary Studies of Residence Time

Acidolysis occurs through sequential reactions of hydrolysis and triglyceride re-synthesis (esterification) [58]. The hydrolysis and esterification capacity of the biocatalyst was assessed by determining hydrolysis and esterification activities. The hydrolysis and esterification activities of Lipozyme RM IM were 352.57 U g^−1^ and 5543.87 U g^−1^, respectively.

Prior to performing CCRD runs, preliminary tests were carried out to assess the relationship between cycle number and the DI of capric acid in cottonseed oil. In general, FBRs require long reaction times for biotransformation compared with stirred-tank and packed-bed reactors, given the lower amount of immobilized enzyme per volume of substrate [2]. To circumvent this limitation, FBR can be operated in batch mode, with recirculation of the substrate through the bed. Recirculation provides an increase in fluidization and, consequently, in the contact time between substrate and biocatalyst [2,59]. 

The FBR was packed with 3.5 g of Lipozyme RM IM and maintained at 45 °C. The substrate (cottonseed oil and capric acid at a 1:2 molar ratio) was fed continuously at a flow rate of 300 mL h^−1^, 1.5 times the minimum fluidization velocity, with recirculation, for up to 130 min. Samples were collected at cycles 1 and 10, corresponding to 13 and 130 min of reaction, respectively, to assess the DI of capric acid in cottonseed oil. The DI was found to be 3.43 mol% after 1 cycle and 20.09 mol% after 10 cycles of recirculation in FBR. Assuming a linear relationship between the DI and cycle number and knowing that the maximum DI, in the case of MLM triacylglycerols, is 66.67 mol% (2/3 of the total fatty acids composing the triglyceride), we performed CCRD runs with 20 (−1) and 70 (+1) cycles. 

#### 2.4.2. Evaluation of the Influence of Residence Time and Oil/Acid Molar Ratio on the Synthesis of Low-Calorie MLM Triacylglycerols

A 2^2^ CCRD with axial points was applied to determine the experimental conditions that provide the maximum DI in FBR. The independent variables were cycle number (residence time) and cottonseed oil/capric acid molar ratio. The CCRD matrix and experimental results are presented in Table 3.

Statistical analysis of experimental data showed that cycle number and oil/acid molar ratio had significant positive effects (*p* < 0.05) on the DI of capric acid in cottonseed oil. In other words, an increase in these variables resulted in an increase in the production of modified triacylglycerols. Similar findings were reported by Choi et al. [60]; the authors investigated the production of structured lipids and found that the concentration of MLM lipids increased significantly with the increase in oil/capric acid molar ratio from 1:3 to 1:5. In acidolysis reactions, where fatty acids are esterified to glycerol, an excess of fatty acids is beneficial because these compounds shift the chemical balance of the reaction toward product formation, resulting in a high DI [9]. However, such behavior occurs up to a certain limit; an excessive amount of fatty acids can lead to acidification of the microaqueous layer at the active site of lipases, causing denaturation [61,62]. In acidolysis reactions with long-chain fatty acids, such as C13:0 and C17:0, enzyme inhibition problems are not as frequent as when short-chain fatty acids (C5:0 and C9:0) are used [60,63].

It is also worth mentioning that an increase in oil/acid molar ratio, that is, an excess of capric acid, can be beneficial due to the increase in reagent availability, enhancing the probability of its esterification to triacylglycerol and causing a shift in esterification equilibrium. Enzymatic esterification is controlled thermodynamically, which contributes to the formation of a stagnate chemical equilibrium [61,64]. A strategy to shift the equilibrium is to produce an excess of one of the substrates [61]. 

A higher cycle number provides an increase in medium recirculation, providing better fluidization and prolonged contact between enzyme and substrate. Thus, the feed flow rate, which is inversely proportional to residence time, becomes independent of fluidization velocity and bed height [2,65]. Studies on enzymatic syntheses in FBR commonly employ long reaction times, such as 24 h [66] and 48 h [40].

Using DI values, we estimated the regression coefficients and constructed first-order models. The mathematical model describing the DI of capric acid (y) as a function of oil/acid molar ratio (X_1_) and cycle number (X_2_) is described in Equation (4).

y = 29.6 + 4.67X_1_ + 6.28X_2_ (4)

For the construction of the model (Equation (9)), only significant parameters were considered, that is, those with *p* < 0.05 and F-values greater than the F-critical (2.9). In this case, the significant parameters were the linear terms of oil/acid molar ratio (X_1_) and cycle number (X_2_). The quadratic terms of both variables and their interaction were not significant, with *p* > 0.05 and F-value < F-critical; therefore, these parameters were excluded from the model. The positive linear effects of cycle number (X_1_) and oil/acid molar ratio (X_2_) indicate that DI tends to increase linearly with increasing X_1_ and X_2_.

**Figure 4:**
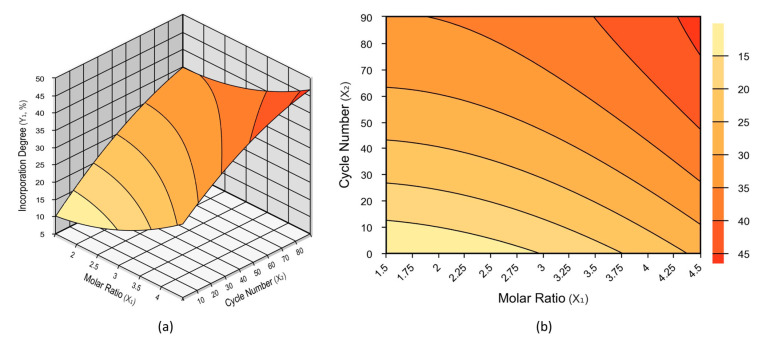
Response surface (**a**) and 2D-Contour (**b**) plots of the degree of incorporation (%, *w*/*w*) of capric acid as a function of cottonseed oil/capric acid molar ratio and cycle number (as a proxy for residence time) in a fluidized bed reactor using Lipozyme RM IM as catalyst.

Analysis of variance (see Supplementary Material, Figures S2 and S3) was performed to assess the adequacy of the adjusted model. The R^2^ was 0.9128, demonstrating that the model explained 91.28% of the variance in DI. The F-value was 10.46, higher than the F-critical (2.9), indicating that the first-order model (Equation (10)) successfully described capric acid incorporation as a function of oil/acid molar ratio and cycle number. Furthermore, the relative error, that is, the variation between observed (experimental) and predicted values, did not surpass 13.8% (Table 3). The low relative errors indicate a good correlation between the mathematical model (Equation (4)) and experimental values in the studied range. The Equation (4) was used to generate response surface plots, depicted in Figure 4.

Figure 4 shows that the maximum DI is achieved by increasing the cycle number (mean residence time) and oil/acid molar ratio (excess capric acid). The CCRD runs that provided the highest DI (~40 mol%) were runs 4 and 6, carried out using high oil/acid molar ratios and a high cycle number. Run 4 was performed using a 1:4 oil/acid molar ratio and 70 cycles (15.17 h), resulting in a capric acid DI of 40.88 mol%. Run 6 was performed using a 1:3 oil/acid molar ratio and 80 cycles (17.34 h), affording a DI of 40.78 mol%. The runs that afforded the lowest DIs were 1 (19 mol%) and 7 (20.83 mol%), which were performed using oil/acid molar ratios of 1:2 and 1:1.59 and fewer cycles, namely 20 (4.34 h) and 45 (9.75 h), respectively. 

The peroxide value was determined at the end of each run (see Supplementary Material Table S2). The peroxide value is an indicator of oil quality and storage stability, providing information on the degree of oil oxidation, as peroxides are oxidation products. The peroxide values of the reactions were below the regulatory threshold of 10 mEq kg^−1^ [44,55], namely of 9.36 ± 0.29 and 6.33 ± 0.15 mEq kg^−1^ for runs 4 and 6, respectively.

On the basis of experimental, model, and response surface results (Figure 4), as well as peroxide values, the best conditions for the synthesis of low-calorie MLM triacylglycerols were determined as an oil/acid ratio of 1:4 and 80 cycles. The independent variable that most influenced the incorporation of capric acid was cycle number (Equation (4)). Thus, the condition of the axial point (+1.41, 80 cycles) was chosen for model validation and the study of operational stability. Oil/acid molar ratio also had a positive impact on capric acid incorporation, but its effect was weaker; thus, it was decided to use its positive factorial point (+1, 1:4 oil/acid) for the study of MLM triglyceride production using Lipozyme RM IM.

**Figure 5:**
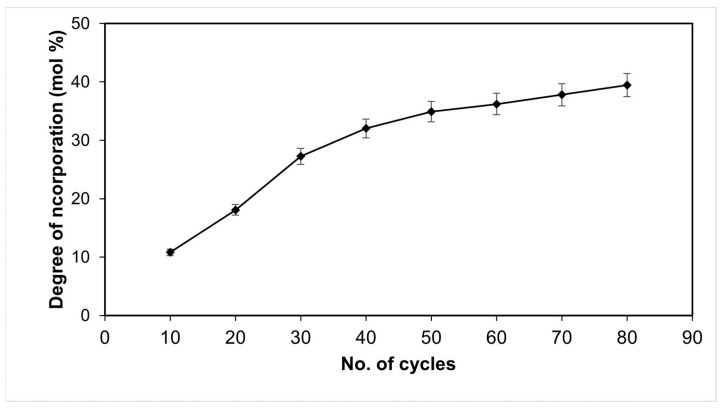
Effect of cycle number on the degree of incorporation (mol%) of capric acid in cottonseed oil using a 1:4 oil/acid molar ratio and Lipozyme RM IM as catalyst in a fluidized bed reactor.

Model validation was performed by repeating the acidolysis reaction under the optimal conditions (1:4 oil/acid molar ratio, 80 cycles) and calculating the relative error between experimental and predicted values. The validation experiment afforded a DI of 40.45 mol% (Figure 5). The predicted DI was 43.32 mol%, resulting in a relative error of 7.36 mol%. This value is close to the relative errors between CCRD runs and the predicted value (Table 3). The low relative error demonstrates that the mathematical model (Equation (4)) is valid; that is, it showed good agreement with experimental values.

The DI (~40 mol%) obtained here, in about 17 h of reaction, is relevant and shows the great potential of the FBR system for the synthesis of MLM triacylglycerols. Bassan et al. [9], in studying the production of low-calorie MLM triacylglycerols in a batch stirred-tank reactor, obtained a DI of 34.53 mol% for capric acid in grape seed oil in 24 h of reaction at 45 °C, using a fatty acid/oil molar ratio of 2:1. Working with a batch stirred-tank reactor and Lipozyme RM IM for the production of structured lipids, Nunes et al. [14] obtained a DI of capric acid in olive oil of 27.1 mol% after 24 h of reaction. Runs were conducted at 45 °C using 5% *w*/*w* Lipozyme RM IM and 1:2 olive oil/capric acid molar ratio. Hamam and Budge [35] produced structured lipids from fish oil and capric acid and achieved a DI of 31.8 mol% in a packed-bed reactor using Lipozyme RM IM, 1:2.7 oil/acid molar ratio, and 12 h of reaction. 

It is known that enzyme-catalyzed synthesis of structured lipids is slow. A strategy to increase the reaction rate is to use reactors that enhance heat and mass transfer. Reactors such as FBR, high-pressure, and batch stirred tank (BSTR), associated with ultrasound or microwave treatment, can reduce reaction times and provide higher reaction yields given the improvement in heat and mass transfer [10,67]. The most widely used reactors for the production of structured lipids are batch stirred-tank reactors (BSTR) [9,32,68] and packed-bed reactors (PBR) [16,35,37]. These reactors have some limitations. BSTR, for instance, have high downtime between production batches and generally low operational stability, given the shear stress exerted on enzymes by mechanical agitation [2,41]. PBR, on the other hand, have low mass transfer and greater possibility of preferential pathway formation, compression, and column clogging [10]. FBRs are considered a hybrid of stirred-tank and packed-bed reactors, showing promise for the production of low-calorie lipids, given their reduced mechanical damage to immobilized enzymes and low-pressure drops compared with packed-bed reactors [2,69].

Another advantage of FBRs is their high mass transfer, provided by the high feed rate, which generates turbulence inside the reactor. As a result, better inter- and intraparticle diffusivity is achieved, leading to the high conversion of substrates [2]. In the transesterification of viscous oils using immobilized lipases, FBRs had better mixing capacity than packed-bed reactors, increasing the productivity of the system [70]. 

### 2.5. Operational Stability

Immobilized lipases provide the possibility of recovering enzymes, optimizing the operational costs of bioprocesses [71]. Strategies to make enzyme-catalyzed processes industrially competitive include the use of low-cost biocatalysts with high enzyme activity and high operational stability [72] Here, Lipozyme RM IM was recovered after acidolysis under the optimal conditions (80 cycles and oil/acid molar ratio of 1:4) and its hydrolytic activity was assessed. The hydrolytic activity of lipase before and after acidolysis was 352.57 ± 3.67 and 350.91 ± 5.96 U g^−1^, respectively. The catalyst lost only 0.47% of its initial activity after 80 cycles, allowing its reuse. Compared with other reaction systems such as stirred-tank reactors (STR), FBRs expose enzymes to mild conditions, given the absence of mechanical agitation, preserving enzyme activity [2,68]. 

From the results of hydrolytic activity, we calculated the thermal deactivation constant (*k*_d_) (Equation (4)) and half-life (*t*_1/2_) (Equation (5)). The 80 cycles corresponded to 17.34 h of reaction. The *k*_d_ value was 2.72 × 10^−4^ h^−1^ and the *t*_1/2_ 2545.78 h. Such results confirm the good stability and high reuse capacity of Lipozyme RM IM in acidolysis reactions of cottonseed oil with capric acid. In a PBR, Lipozyme RM IM was found to have a *k*_d_ of 6.1 × 10^−3^ h^−1^ and *t*_1/2_ of 235.63 h, in the synthesis of MLM triacylglycerols by enzymatic acidolysis of grape seed oil with capric acid [16]. When used for the synthesis of 2-ethylhexyl oleate catalyzed by *C. antarctica* lipase immobilized on magnetic poly(styrene-*co*-divinylbenzene) particles in FBR, the biocatalyst had a *k*_d_ of 4.04 × 10^−4^ h^−1^ and *t*_1/2_ of 1716 h. Weaver et al. [73] assessed the operational stability of Lipozyme RM IM in the synthesis of structured lipids with different acyl donors in a batch stirred-tank reactor. When oleic acid was used, the lipase did not suffer a decrease in activity for 10 cycles of 23 h, whereas, when omega-3 polyunsaturated fatty acids were used, the lipase showed linear deactivation, with a *t*_1/2_ of 276 h. The operational stability of enzymes is strongly dependent on medium composition and, more specifically, in the case of lipid modification, on the acyl donor used [61]. Another important factor for the maintenance of enzymatic activity is the type of support in which the enzyme is immobilized and its interaction with the reaction substrates and products [18]. In the enzymatic synthesis of eugenol esters using packed bed microreactors, regarding operational stability, Lipozyme RM IM was shown to be more stable for the 26 h of the study, while Novozym 435 showed a 10% higher loss in conversion over the same period of time [18].

### 2.6. sn-2 Position Analysis of Modified Triacylglycerols

Modified triacylglycerols were evaluated to assess which compounds were occupying the *sn*-2 position. This analysis allows determining whether capric acid was incorporated at positions 1 or 3 of triacylglycerol, reflecting the MLM lipid of interest. The results of *sn*-2 position analysis of cottonseed oil (before acidolysis) and the reaction product are shown in Table 4.

The predominant fatty acids at *sn*-2 of modified triacylglycerols were linoleic (65.68%) and oleic (26.28%) acids. Incorporation of capric acid at *sn*-2 was minimal (1.8%). Lipozyme RM IM is *sn*-1,3-regioselective; however, despite its regiospecificity, during acidolysis, capric acid may be incorporated at *sn*-2, resulting from acyl migration [73]. Acyl migration is influenced by high temperature, reaction time, acyl chain length, water activity of the reaction medium, reaction system, and lipase regioselective, among other factors [74,75]. In this study, the low incorporation of capric acid at *sn*-2 may be attributed to the mild reaction temperature (45 °C) and the use of a *sn*-1,3-regioselective lipase.

In view of the results of the *sn*-2 position analysis (Table 4), it is possible to affirm that capric acid was incorporated mainly at *sn*-1 and -3 of modified grape seed oil, resulting in an MLM triglyceride. The MLM triglyceride is composed of long-chain fatty acids (18 carbons) at position 2 and medium-chain fatty acids (10 carbons) at positions 1 and 3.

## 3. Materials and Methods

### 3.1. Materials

Experiments were performed using Lipozyme RM IM (lipase from R. miehei), kindly donated by Novozymes^®^ (Araucária, PR, Brazil). Cottonseed (*Gossypium* spp.) oil (Distriol^®^, Ipiranga, SP, Brazil) and capric acid (Merck^®^, Darmstadt, Germany) were used as raw materials. All other reagents were of analytical grade.

### 3.2. Analytical Methods

#### 3.2.1. Determination of Esterification Activity

The esterification activity of the commercial lipase preparation (200 mg of enzyme) was assessed by esterifying oleic acid and ethanol at a molar ratio of 1:1, 40 °C, and 200 rpm. Oleic acid consumption was determined by titration with 0.05 M sodium hydroxide [76]. One unit of esterification activity was defined as the amount of enzyme required to esterify 1 µmol of oleic acid per minute under the above-mentioned conditions. Results are expressed in µmol g^−1^ min ^−1^ (U g^−1^).

#### 3.2.2. Determination of Hydrolytic Activity

Hydrolytic activity was determined by the back titration method using hydrochloric acid, olive oil, and gum arabic [36]. One unit of hydrolytic activity was defined as the amount of enzyme required to hydrolyze 1 µmol of fatty acid per minute of reaction. The results are expressed in µmol g^−1^ min^−1^ (U g^−1^).

#### 3.2.3. Characterization of Raw Materials

The acid value (method Ca 5a-40) and peroxide value (method Cd 8b-90) of cottonseed oil were determined according to AOCS methods [77] and expressed in mg KOH g^−1^ and mEq kg^−1^, respectively. 

#### 3.2.4. Determination of Fatty Acid Profile

Samples were first neutralized with a hydroalcoholic solution (30% *v*/*v* ethanol) containing 0.8 M potassium hydroxide [78] and then subjected to methylation [79] under sequential acid and alkaline conditions. Fatty acids were analyzed by gas chromatography according to method Ce 2-66 (AOCS, 2004). A PerkinElmer^®^ gas chromatograph equipped with a split injector, flame ionization detector, and Supelcowax capillary column (30 m × 0.32 mm, 0.50 µm) was used. Injector and detector temperatures were maintained at 250 °C. Samples were injected in split mode (1:10). Nitrogen was used as carrier gas at a flow rate of 0.5 mL min^−1^. The column temperature was started at 60 °C, raised to 210 °C at 20 °C min^−1^, maintained at 210 °C for 7 min, increased to 250 °C at 20 °C min^−1^, and held at this temperature for 25 min. Fatty acids were identified and quantified by comparison of their peak areas with those of internal and external standards. The external standard was Supelco 37 Component FAME mix C4–C24 (methyl butyrate–methyl cis-15-tetracosenoate) (Sigma–Aldrich, Darmstadt, Germany). The internal standard was methyl myristate (C14) (Sigma–Aldrich, Darmstadt, Germany).

#### 3.2.5. Determination of the Degree of Incorporation (DI)

The DI (mol%) of fatty acids was calculated according to Equation (5) [80]:

(5) $$\mathrm{DI}=\frac{\mathrm{MFA}}{\mathrm{TFA}}\times 100$$

where MFA is the number of mol of medium-chain fatty acids (C10:0) in triglyceride molecules and TFA is the total number of moles of fatty acids in triglyceride molecules.

#### 3.2.6. Analysis of *sn*-2 Fatty Acids

Profile analysis of fatty acids at the sn-2 position of triacylglycerol molecules was performed according to the hydrolysis method using an *sn*-1,3-regioselective lipase from porcine pancreas (PPL). A 100 mg aliquot of PPL was weighed into a centrifuge tube and 1 mL of Tris-HCl buffer (pH 8), 0.250 mL of bile salt, 0.1 mL of calcium chloride, and 100 mg of modified triglyceride oil were added. The reaction was conducted at 40 °C in a water bath for 30 min and stopped by the addition of 1 mL of 6 M HCl and 1 mL of ether. The mixture was centrifuged at 5000 rpm for 10 min, and the upper phase was collected and applied to a thin-layer chromatography plate (silica gel containing a 254 nm fluorescence indicator). The plate was taken to a glass tank containing a solution of hexane, ether, and acetic acid (70:30:1). The plate was sprayed with an alcoholic solution of 0.2% 2,7-dichlorofluorescein. The monoglyceride band was scraped and analyzed by gas chromatography, as described in Section 3.2.4.

#### 3.2.7. Statistical Analysis

The effects of independent variables on the results of central composite rotatable design (CCRD) experiments were assessed using Statistica software version 12.0 (Statsoft Inc., Tulsa, OK, USA). The level of significance was set at *p* < 0.05. 

### 3.3. Instrumental Methods

#### 3.3.1. Physical Characterization of Reaction Mixtures at Different Molar Ratios

The viscosity of reaction mixtures prepared using different molar ratios of cottonseed oil/capric acid (1:2, 1:3, and 1:4) was measured on a Brookfield^®^ (Toronto, ON, Canada) DV-III Ultra rheometer.

#### 3.3.2. Hydrodynamic Characterization of the FBR

The hydrodynamic behavior of the reaction mixture in the reactor was studied by measuring the axial dispersion [37]. A pulse tracer test was performed using the fat-soluble dye solution Mix Verde^®^ (purchased from a local market Araraquara, SP, Brazil) at a concentration of 1 g mL^−1^. The system consisted of a jacketed column reactor (18.70 cm height and 2 cm diameter) containing approximately 4 g of lipase and a 1:2 (mol/mol) mixture of cottonseed oil/capric acid, connected to an ultrathermostatic bath (Lucadema^®^, São José do Rio Preto, SP, Brazil) at 45 °C and a positive displacement pump (Grundfos^®^, Bjerringbro, Denmark). The test was run continuously for 90 min. Aliquots (2 mL) of the reaction medium were collected every 3 min, in duplicate, and read at 415 nm using a GENESYS 10S UV-Vis spectrophotometer (Thermo Fisher Scientific, San Jose, CA, USA). The residence time distribution function was calculated as shown in Equation (6):

(6) $$E\left(t\right)=\frac{C\left(t\right)}{\int_{0}^{\infty }C\left(t\right)dt}$$

where *E*(*t*) is the residence time distribution function and *C*(*t*) is the tracer concentration at a given time. The denominator term represents the area under the tracer concentration curve (given by the integral) as a function of time.

The mean residence time (*t*_m_) was calculated according to Equation (7).

(7) $$t_{m}=\int_{0}^{\infty }E\left(t\right)\times tdt$$

The integrals of Equations (6) and (7) were resolved using Origin software version 8.0 (OriginLab serial GA3S5-6089-7173339, Northampton, MA, USA). For analysis of preferential flow pathways, the results of space time (theoretical, Equation (8)) were compared with the mean residence time (experimental, Equation (7)).

(8) $$\tau =\frac{V_{u}}{Q_{v}}$$

where *Q*_V_ represents the volumetric flow rate and *V*_u_ the useful volume of the fluidized bed.

#### 3.3.3. Minimum Fluidization Velocity as a Function of Biocatalyst Mass

The minimum fluidization velocity was determined according to Kunii and Levenspiel [56]. Tests were conducted in a jacketed column reactor (18.70 cm height and 2.00 cm diameter) containing a 1:2 (mol/mol) mixture of cottonseed oil/capric acid as reaction medium and 1.5 g of biocatalyst. The temperature was maintained at 45 °C. The flow rate was gradually increased from 100 to 300 mL h^−1^ in increments of 10 mL h^−1^ by using a recirculation pump (Grundfos^®^, Bjerringbro, Denmark). Then, the flow rate was gradually decreased, and the bed height provided by each flow rate was assessed visually. Results were plotted on a graph of bed height (cm) as a function of flow rate (mL h^−1^).

#### 3.3.4. Synthesis of MLM Triacylglycerols

Acidolysis of cottonseed oil and capric acid was conducted in an FBR reactor (18.70 cm height and 2.0 cm diameter) with recirculation. The jacketed reactor was coupled to a thermostatic bath, which allowed us to maintain the system temperature at 45 °C. The substrate was fed into the system at a flow rate of 300 mL h^−1^ by using a positive displacement pump connected to the reactor. The column was filled with 3.5 g of biocatalyst. Cycle number (used as a proxy for residence time) and substrate molar ratio were varied according to the conditions described in Section 3.3.5. Aliquots of the reaction medium were collected at predetermined intervals and analyzed for fatty acid composition (Section 3.2.4) and DI (Section 3.2.5).

#### 3.3.5. Effect of Cycle Number and Substrate Molar Ratio on MLM Triacylglycerols Synthesis

A CCRD was used to investigate the influence of cycle number (residence time) and substrate molar ratio on the incorporation of capric acid in cottonseed oil. The design was a 2^2^ factorial with 4 axial points and 3 replications of the center point, totaling 11 runs (Table 5). All experiments were performed in an FBR, as described in Section 3.3.4.

#### 3.3.6. Analysis of Operational Stability

For the analysis of operational stability, the immobilized lipase used in each reaction was collected and washed with hexane added with anhydrous sodium sulfate as desiccant. After washing, the biocatalyst was filtered under vacuum and stored for 6 h in a desiccator at 20 °C. The hydrolytic activity of the recovered derivative was quantified according to the procedures described in Section 3.2.2. The values of hydrolytic activity before and after acidolysis were used to calculate the thermal deactivation constant (*k*_d_) and half-life (*t*_1/2_), using Equations (9) and (10), respectively [81]:

(9) $$\ln A=\ln A_{0\;}-k_{d}t$$

(10) $$t_{1/2}=\frac{-\ln\left(0.5\right)}{k_{d}}$$

where *A*_0_ is the initial enzyme activity (U g^−1^), *A* is the enzyme activity after the acidolysis reaction (U g^−1^), *k*_d_ is the thermal deactivation constant (h^−1^), and t is the reaction time (h).

## 4. Conclusions

Lipozyme RM IM afforded relevant values (~40 mol%) of capric acid incorporation (C10) in cottonseed oil in an FBR. The independent variables studied using a CCRD were residence time (cycle number) and oil/acid molar ratio. Both factors were found to positively influence capric acid incorporation. The optimal conditions for the production of MLM triacylglycerols in FBR were found to be a 1:4 oil/acid molar ratio and 80 cycles (17.34 h) at 45 °C. FBR proved to be an interesting and efficient alternative for acidolysis of cottonseed oil with capric acid. The use of Lipozyme RM IM in an FBR reaction system under optimal process conditions resulted in high operational stability (*k*_d_ = 2.72 × 10^−4^ h^−1^ and *t*_1/2_ = 2545.78 h), confirming the high reuse capacity of lipases in acidolysis reactions. The search for new reactor configurations, such as FBR, is important for the implementation of enzymatic production systems for low-calorie MLM triacylglycerols in the food industry. In order to obtain higher incorporation of capric acid in cottonseed oil, future studies can be carried out evaluating FBR associated with ultrasound or microwave treatment, strategies that can reduce reaction times and provide higher reaction yields.

## Figures and Tables

**Table 1:** Fatty acid profile of cottonseed oil.

Fatty Acid	Concentration (%, *w*/*w*)
Palmitic acid (C16:0)	11.27
Stearic acid (C18:0)	4.83
Oleic acid (C18:1n9)	26.94
Linoleic acid (C18:2n6)	50.24
Linolenic acid (C18:3n3)	4.28
Gamma-linolenic acid (C18:3n6)	0.22
Unidentified fatty acids	2.22

**Table 2:** Density of reaction mixtures prepared using different molar ratios of cottonseed oil to capric acid.

Cottonseed Oil/Capric Acid Molar Ratio	Density (g mL^−1^)
1:2	0.9215 ^a^ ± 0.0416
1:3	0.9108 ^a^ ± 0.0252
1:4	0.9079 ^a^ ± 0.0920

Values are the mean ± standard deviation of triplicate determinations. Means followed by the same letter (^a^) are not significantly different by Tukey’s test (*p* < 0.05).

**Table 3:** Central composite rotatable design matrix (2^2^ full factorial design with axial points) for evaluation of the effects of cottonseed oil/capric acid molar ratio and cycle number on the degree of incorporation of capric acid into triacylglycerols in a fluidized bed reactor.

Run	Oil/Acid Molar Ratio ^1^	Cycle Number ^1^	Reaction Time (h)	Experimental Degree of Incorporation ^2^ (mol%)	Predicted Degree of Incorporation ^3^ (mol%)	Relative Error ^4^ (%)
1	−1 (1:2)	−1 (20)	4.34	19	18.65491	1.842105
2	−1 (1:2)	1 (70)	15.17	25.74	27.98653	8.74126
3	1 (1:4)	−1 (20)	4.3	35.7	31.22069	12.57703
4	1 (1:4)	1 (70)	15.17	40.88	40.55231	0.807241
5	0 (1:3)	−1.41 (10)	2.17	22.84	23.02482	0.76751
6	0 (1:3)	1.41 (80)	17.34	40.78	36.1824	11.26851
7	−1.41 (1:1.59)	0 (45)	9.75	20.83	20.74474	0.407105
8	1.41 (1:4.41)	0 (45)	9.75	33.79	38.46248	13.8053
9	0 (1:3)	0 (45)	9.75	29.67	29.60361	0.235929
10	0 (1:3)	0 (45)	9.75	30.61	29.60361	3.299575
11	0 (1:3)	0 (45)	9.75	28.54	29.60361	3.71409

^1^ Independent variables are presented as coded and actual (in parentheses) values. ^2^ Response variable. ^3^ Calculated according to Equation (4). ^4^ $\mathrm{Relative}\;\mathrm{error}=\left(\frac{\mathrm{Experimental}\;\mathrm{value}\;-\;\mathrm{Predicted}\;\mathrm{value}}{\mathrm{Experimental}\;\mathrm{value}}\right)\times 100.$

**Table 4:** Composition of *sn*-2 fatty acids in cottonseed oil and modified triglyceride oil obtained from a 1:4 mixture of cottonseed oil and capric acid after 80 reaction cycles.

Sample	Fatty Acid	Concentration (%, *w*/*w*)
Cottonseed oil	Palmitic acid (C16:0)	2.87
	Stearic acid (C18:0)	0.79
	Oleic acid (C18:1n9)	32.24
	Linoleic acid (C18:2n6)	60.78
	Linolenic acid (C18:3n3)	3.32
Modified triglyceride oil	Capric acid (C10:0)	1.80
	Palmitic acid (C16:0)	1.06
	Stearic acid (C18:0)	0.48
	Oleic acid (C18:1n9)	26.28
	Linoleic acid (C18:2n6)	65.68
	Linolenic acid (C18:3n3)	4.7

**Table 5:** Real and coded values of independent variables analyzed using a central composite rotatable design.

Independent Variable	Level				
	−1.41	−1	0	+1	+1.41
Cottonseed oil/capric acid molar ratio	1.59	2	3	4	4.41
Cycle number	10	20	45	70	80

## Data Availability

Samples of the compounds and all data are available from the authors.

## Supplementary Materials

The following supporting information can be downloaded at: https://www.mdpi.com/article/10.3390/molecules28145384/s1, Table S1: Absorbance measurements of fat-soluble dye for construction of analytical curve and determination of residence time of FBR. Figure S1: The analytical curve for determination of residence time in the hydrodynamic characterization of FBR. Figure S2: Analysis of variance (ANOVA) of the central composite rotatable design (2^2^ full factorial design with axial points) for evaluation of the effects of cottonseed oil/capric acid molar ratio and cycle number on the degree of incorporation of capric acid into triacylglycerols in a FBR; Figure S3: Regression coefficients of the central composite rotatable design (2^2^ full factorial design with axial points) for evaluation of the effects of cottonseed oil/capric acid molar ratio and cycle number on the degree of incorporation of capric acid into triacylglycerols in a FBR; Table S2: Peroxide value determined at the end of each run of Central Composite Rotatable Design (2^2^ full factorial design with axial points) in the FBR.
