# Supplementary material for: Solvent-Free Enzymatic Synthesis of Dietary Triacylglycerols from Cottonseed Oil in a Fluidized Bed Reactor
Source: Molecules. 2023 Jul 13;28(14):5384. doi: 10.3390/molecules28145384 (PMC10384263; doi:10.3390/molecules28145384)
Supplement: Supplementary file 1 [file molecules-28-05384-s001.zip › molecules-2476096-supplementary.pdf]

Supplementary Materials

Solvent-Free Enzymatic Synthesis of Dietary Triacylglycerols from Cottonseed Oil in a Fluidized Bed Reactor

Daniela Remonatto [1], Núbia Santaella [1], Lindomar Alberto Lerin [2,*], Juliana Cristina Bassan [1,3], Marcel Otávio Cerri [1] and Ariela Veloso de Paula [1,*]

[1] Department of Bioprocess Engineering and Biotechnology, School of Pharmaceutical Sciences, São Paulo State University (UNESP), Araraquara 14800-903, SP, Brazil; d.remonatto@unesp.br (D.R.); nubiasantaella@gmail.com (N.S.); juliana.bassan@unesp.br (J.C.B.); marcel.cerri@unesp.br (M.O.C.)
[2] Department of Chemistry, Pharmaceutical and Agricultural Sciences, University of Ferrara (UNIFE), Via Luigi Borsari, 46, 44121 Ferrara, Italy
[3] State Center for Technological Education Paula Souza, Faculty of Technology of Barretos (FATEC), Barretos 14780-060, SP, Brazil
* Correspondence: lrnldm@unife.it (L.A.L.); ariela.veloso@unesp.br (A.V.d.P.); Tel.: +39-532-455418 (L.A.L.); +55-16-3301-4648 (A.V.d.P.)

Table S1. Absorbance measurements of fat-soluble dye for construction of analytical curve and determination of residence time of FBR.

	Absorbance (λ=415nm)				
**Dye concentration (g/L)**	**Abs1**	**Abs2**	**Abs3**	**Abs means**	**Standard deviation**
0.10	0.079	0.078	0.073	0.077	0.0032
0.20	0.167	0.166	0.157	0.163	0.0055
0.30	0.282	0.290	0.270	0.281	0.0101
0.40	0.316	0.322	0.345	0.328	0.0153
0.50	0.454	0.403	0.388	0.415	0.0346
0.60	0.509	0.492	0.514	0.505	0.0115
0.70	0.623	0.656	0.657	0.645	0.0193
0.80	0.741	0.705	0.699	0.715	0.0227

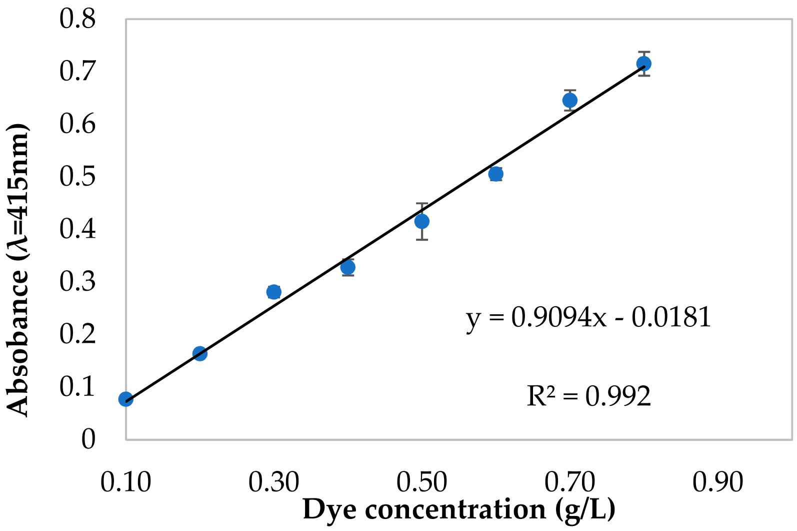

Figure S1. The analytical curve for determination of residence time in the hydrodynamic characterization of FBR.

	ANOVA; Var.:Var3; R-sqr=,91277; Adj:,82554 (Spreadsheet10) 2 factors, 1 Blocks, 11 Runs; MS Residual=9,740095 DV: Var3				
Factor	SS	df	MS	F	p
(1)Molar Ratio(L)	314,8583	1	314,8583	32,32600	0,002346
Molar Ratio(Q)	5,1621	1	5,1621	0,52999	0,499262
(2)Number of Cycles(L)	173,6398	1	173,6398	17,82732	0,008310
Number of Cycles(Q)	9,5182	1	9,5182	0,97722	0,368279
1L by 2L	0,6084	1	0,6084	0,06246	0,812588
Error	48,7005	5	9,7401		
Total SS	558,3130	10			

Figure S2. Analysis of variance (ANOVA) of the central composite rotatable design (2^2 full factorial design with axial points) for evaluation of the effects of cottonseed oil/capric acid molar ratio and cycle number on the degree of incorporation of capric acid into triglycerides in a FBR.

	Regr. Coefficients; Var.:Var3; R-sqr=,91277; Adj:,82554 (Spreadsheet10) 2 factors, 1 Blocks, 11 Runs; MS Residual=9,740095 DV: Var3					
Factor	Regressn Coeff.	Std.Err.	t(5)	p	-95,% Cnf.Limt	+95,% Cnf.Limt
Mean/Interc.	29,60361	1,801838	16,42967	0,000015	24,97184	34,23538
(1)Molar Ratio(L)	6,28289	1,105054	5,68560	0,002346	3,44226	9,12352
Molar Ratio(Q)	-0,95996	1,318617	-0,72800	0,499262	-4,34957	2,42966
(2)Number of Cycles(L)	4,66581	1,105054	4,22224	0,008310	1,82517	7,50644
Number of Cycles(Q)	1,30351	1,318617	0,98854	0,368279	-2,08610	4,69313
1L by 2L	-0,39000	1,560456	-0,24993	0,812588	-4,40128	3,62128

Figure S3. Regression coefficients of the central composite rotatable design (2^2 full factorial design with axial points) for evaluation of the effects of cottonseed oil/capric acid FBR.

Table S2. Peroxide value determined at the end of each run of Central Composite Rotatable Design (2^2 full factorial design with axial points) in the FBR.

Run	Peroxide value (mEq kg^{-1})
1	1.98 ± 0.16
2	5.83 ± 0.58
3	12.14 ± 0.11
4	9.36 ± 0.29
5	7.85 ± 0.35
6	6.33 ± 0.15
7	11.02 ± 0.03
8	3.32 ± 0.29
9	4.37 ± 0.29
10	3.94 ± 0.01
11	3.53 ± 0.60
